# Effect of Ambulatory Oxygen on the Respiratory Pattern during the 6 Min Walking Test in Patients with Interstitial Lung Diseases

**DOI:** 10.3390/biomedicines11071834

**Published:** 2023-06-26

**Authors:** Vittoria Ventura, Magda Viani, Francesco Bianchi, Miriana d’Alessandro, Piersante Sestini, Elena Bargagli

**Affiliations:** Respiratory Diseases Unit, Department of Medical and Surgical Sciences & Neurosciences, University of Siena, 53100 Siena, Italy

**Keywords:** oxygen-therapy, interstitial lung diseases, exercise, six-minute walk test

## Abstract

Introduction: Patients with pulmonary fibrosis experience early oxyhemoglobin desaturation under effort, which limits their ability to exercise and their quality of life. Recent studies have shown that in resting normoxaemic patients who become hypoxemic under exertion, administration of outpatient oxygen significantly improves stress dyspnoea and quality of life. It is unclear how this happens, since oxygen administration does not act directly on dyspnoea, and does not appear to have much effect on the heart rate and pulmonary artery pressure. We tested the hypothesis that correcting the hypoxaemia could reduce the increase in respiratory effort during the 6 min walking test, recording the breathing pattern during administration of oxygen or placebo. Methods: We evaluated 20 patients with fibrotic interstitial lung diseases (17 males and 3 females; mean age 72 ± 2 years; M ± SE) with a resting SpO^2^ ≥92 that fell to ≤88% during the 6 min walk test (6MWT). After first establishing the oxygen flow necessary to prevent desaturation, the patients underwent two further 6MWT, 15–20 min apart, one with administration of medical air and one with oxygen at the same flow, in randomized double-blind order. During the test, SpO^2^, heart rate, respiratory rate, tidal volume and minute ventilation (VE) were recorded, using a Spiropalm spirometer (Cosmed, Rome, Italy). Results: Oxygen saturation during the 6MWT decreased to a minimum value of 82.3% (95% CI 80.1–84.5%) during placebo and to 92% (90.3–93.7%) during oxygen with an average difference of 9.7% (7.8–11.6%, *p* < 0.0001). On the contrary, heart rate showed an increasing trend with walking time reaching a significantly higher maximum rate during placebo, with a difference of 5.4 bpm (2.9–8.7, *p* < 0.005) compared to oxygen. The distance walked was slightly but significantly greater after oxygen by 28 m (2–53, *p* < 0.05) and end of test dyspnoea after placebo by 0.6 points (0.1–1.1, *p* < 0.05). Respiratory rate increased over time, without differences between oxygen and placebo in the first minute of walking, then increasing significantly more during placebo (*p* < 0.0005). With placebo, tidal volume increased rapidly reaching a plateau at about 48% of FVC after 3 min, while with oxygen, the increase was slower, reaching a maximum of about 45% of FVC at the end of the test. Nevertheless, the difference was highly significant (*p* < 0.0005) at all the time points. Minute ventilation also increased significantly with walking time but remained at a highly significant lower level during oxygen than placebo at all the time points. Mean reduction in VE during the test with oxygen compared to placebo was 4.4 L/min (3.9–4.9, *p* < 0.0005). Conclusion: In our ILD patients, administration of outpatient oxygen during walking was related to a reduced increase in heart rate, respiratory rate, tidal volume and minute ventilation necessary to meet increased oxygen requirements, resulting in a lower workload on the cardiovascular system and on respiratory muscles and a consequent reduction in dyspnoea.

## 1. Introduction

Fibrotic Interstitial lung diseases (ILDs) are a heterogeneous group of rare but widespread diseases, characterized by chronic inflammation and progressive fibrosis of the lung parenchyma [1].

Exertional dyspnoea and oxygen desaturation occur frequently from the early stages of the disease [2,3], and ambulatory oxygen has been shown to improve walking distance [4] and quality of life [5] in these patients.

The mechanisms of exertional dyspnoea and desaturation in these diseases are complex, and while the latter appears to be mostly related to pulmonary vascular changes [6,7,8], dyspnoea is mainly attributable to reduced lung compliance and the consequent increase in respiratory effort needed for lung expansion [9].

The two mechanisms probably act in combination during exercise, however, patients with lower lung diffusion capacity tend to present more severe dyspnoea during exercise [10].

Information on the mechanisms through which ambulatory oxygen interfere with these mechanisms, reducing dyspnoea and improving quality of life in these patients, are incomplete and mostly derived from experimental laboratory studies [11], which may not always reflect what happens in real life activities [12].

We tested the hypothesis that by preventing exertional desaturation, ambulatory oxygen could reduce the increase in ventilatory request induced by a common activity such as walking, as performed under controlled conditions during the six minute walking test (6MWT), which has been shown to correlate with many relevant clinical aspects of these diseases [13].

## 2. Methods

In total, 20 patients, 3 females and 12 males (mean age 73 ± 9 years M ± SD, 74% no smokers, 26% ex-smokers, 0% smokers) with diffuse interstitial lung disease (ILD) were recruited for the study: 15 were diagnosed with idiopathic pulmonary fibrosis (IPF, in two cases associated with emphysema), two with idiopathic non-specific interstitial lung disease, two with chronic hypersensitivity pneumonitis and one with suspected idiopathic lymphocytic interstitial lung disease.

All patients gave their written informed consent to participate in the study. The study was approved by the regional ethical review board of Siena, Italy (C.E.A.V.S.E. Markerlung 17,431) and complied with the declaration of Helsinki.

The inclusion criteria of the study were: a definite diagnosis performed according to international guidelines, clinical stability, resting normoxia (SaO^2^ > 92%), and desaturation (SaO^2^ < 88%) during the 6 min walking test. Anthropometric data and pulmonary function test are reported in Table 1. Mean desaturation in the screening 6MWT was 83%.

In a preliminary test, the oxygen flow required to prevent hemoglobin desaturation was determined using a validated procedure (11), using liquid oxygen through a nasal cannula from a portable stroller. The compensatory flows obtained ranged from 1 to 6 L/min, with an average of 2.1 L/min (95% CI 1.7–2.6).

The patients then performed two 6MWT, 20–30 min apart, during administration of oxygen or medical air (placebo), both at the same flow determined in the preliminary test, administered through a nasal cannula in a randomized order. The flows were administered through nasal cannulas connected to the oxygen or medical air tap (located in a room halfway through the corridor, and not visible to the patient or the examiner) by a 16 m plastic tube, which allowed a total walking circuit of 20 m. The tap was controlled by an operator different from the examiner, so that both the patient and the examiner were unaware of what gas was administered in each run.

For the test, the patient, after wearing the nasal prongs, was connected to a Spiropalm spirometer (Cosmed, Rome, Italy) through a silicon face mask sealing the mouth and the nose and fitted with a turbine spirometer. The device records inspiratory and expiratory Tidal Volume (TV) as well as respiratory rate (RR) at 15” intervals. It also contains a pulse oxymeter, connected to a finger probe, that records mean oxygen saturation (SO^2^) and heart rate (HR) over the same intervals [14].

The data are recorded internally and later downloaded on a computer through a USB cable. The display was hidden to the patient and to the examiner during the test, but an internal alarm was set to ring if SO^2^ would fall below 70%, in which case the experiment had to be stopped. The recording was performed for one minute while sitting without any additional gas, one minute after starting gas supplementation, and then during the 6 min of the test.

### Statistical Analysis

To compensate for the bias of the gas flow bypassing the spirometer during inspiration, TV was computed as the mean of inspiratory and expiratory TV over each 15” interval, and Minute Ventilation (VE) was computed as the product of this value, and the RR recorded in the same interval.

The effects of oxygen on the various parameters were evaluated using a mixed model in which the parameter of interest was entered as a dependent variable and the treatment (medical air or oxygen) and the order of treatments as independent fixed variables, while the subject was entered as random-effect variable [15] (Senn “https://onlinelibrary.wiley.com/doi/book/10.1002/0470854596” (accessed on 12 June 2023)). For the analysis, the results were pooled at intervals of one minute, at rest while breathing room air (before any gas was administered), after 1 min of gas administration, and then for every minute during the walking test.

Changes on the effect of oxygen over time were tested in similar models using the difference in the relevant parameter between the two treatments at each time point as the dependent variable and time (starting at 15”, the first time point during walking), as a continuous variable.

A *p*-value lower than 0.05 for a two-tail distribution was considered statistically insignificant. All the analyses were performed using STATA version 17 for Windows (StataCorp, College Station, TX, USA).

## 3. Results

**Figure 1:**
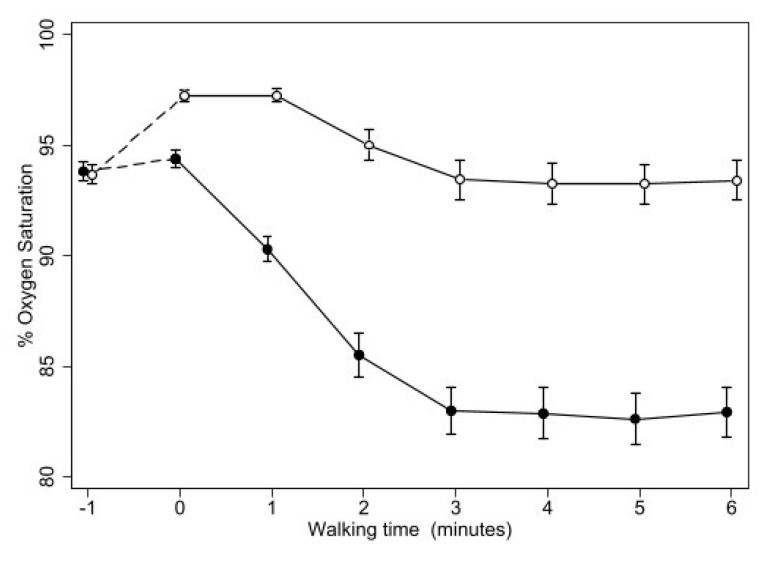
Changes of mean oxygen saturation during 6MWT with oxygen (open circles) or placebo (closed circles). Bars represent the SE. Except for the first point (breathing room air while wearing the mask), the difference between the two treatments was highly significant (*p* < 0.0005) at all the time points.

All patients completed the study, and only one test (during placebo) had to be stopped because of excessive oxygen desaturation. Table 2 reported the data of 6MWT. During placebo, oxygen saturation decreased to values lower than 88% in all the patients, while during oxygen, this occurred only in two patients (in one decreasing to 87–86% for less than a minute between two and three minutes from starting, in one other decreasing below 88% for all the time after two minutes of walking, reaching a minimum value of 84%). Nevertheless, SO^2^ was higher during oxygen than placebo in all the patients at each time point after starting treatment (Figure 1), with a mean difference increasing from 8 to 13% from the 1st to the 6th minute of walking (*p* < 0.001 at each time point and for trend over time).

The average distance covered during the 6 min walking test and dyspnoea at the end of test were slightly but significantly better on oxygen than on placebo, while end of test fatigue was non affected. However, most patients complained that the discomfort caused by wearing the mask was such to impair their estimate of dyspnoea or fatigue. Maximum heart rate and minimum oxygen saturation during the test were markedly improved on oxygen compared to placebo.

**Figure 2:**
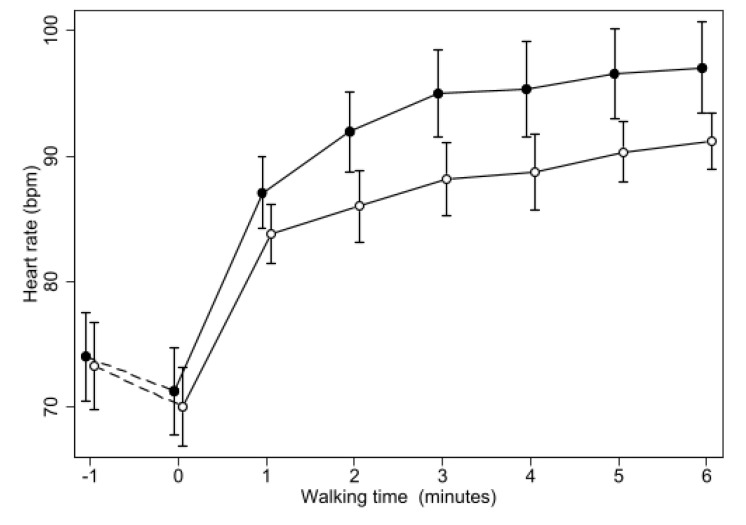
Changes of mean heart rate during 6MWT with oxygen or placebo. Same symbols as in Figure 1. The difference between the two treatments was highly significant (*p* < 0.0005) at all the time points from minute 1 of the walking test.

Heart rate slightly decreased in resting conditions on both occasions, but was not affected by the treatments at rest. Whilst walking, HR rapidly increased during both treatments, but less so during oxygen than placebo, reaching a maximum of 99.5 ± 3.2 bpm (M ± SE) on placebo and of 94.5 ± 2.3 bpm on oxygen, with the difference reducing over time (*p* = 0.001) but resulting highly significant (*p* < 0.001) at every minute during walking (Figure 2).

**Figure 3:**
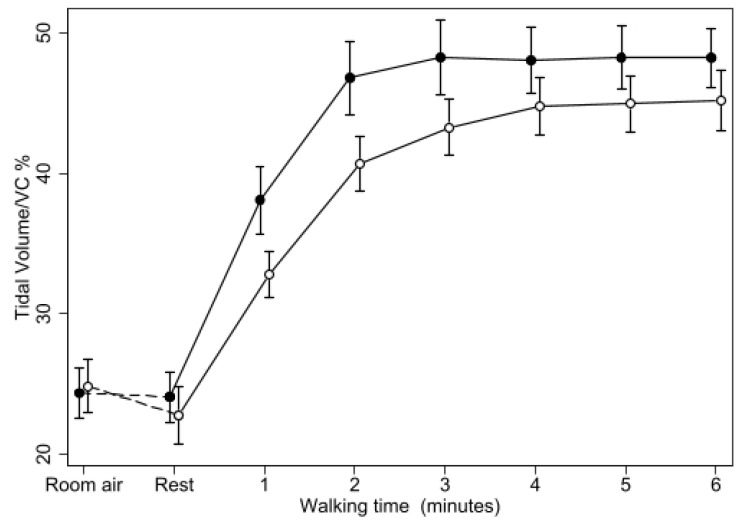
Changes of Tidal Volume (expressed as percentage of FVC) during 6MWT with oxygen or placebo. Same symbols as in Figure 1. The difference between the two treatments was highly significant (*p* < 0.0001) at all the time points from minute 1 of the walking test.

Tidal volume was not affected by the treatments at rest. During placebo, TV did rapidly increase over the first two minutes of walking, remaining then relatively stable for the rest of the test at mean values of around 1.1 L, corresponding to about 48% of FVC (Figure 3).

During oxygen, TV did increase more slowly than on air, reaching a maximum only after 4 min, remaining stabilized afterwards at mean values of about 1 L, still lower than during placebo. The difference in TV between the two treatments was therefore larger in the first minute, when it reached 131 mL (106–159), and decreased in the last minutes of the test, resulting to on average 68 mL (54–81) over the last three minutes. Accordingly, the difference in TV between the two treatments was found to reduce with time of walking (*p* = 0.0005), but it remained highly significant (*p* < 0.0005) at all the time points. Similar results were obtained when using inspiratory or expiratory TV rather than their mean.

**Figure 4:**
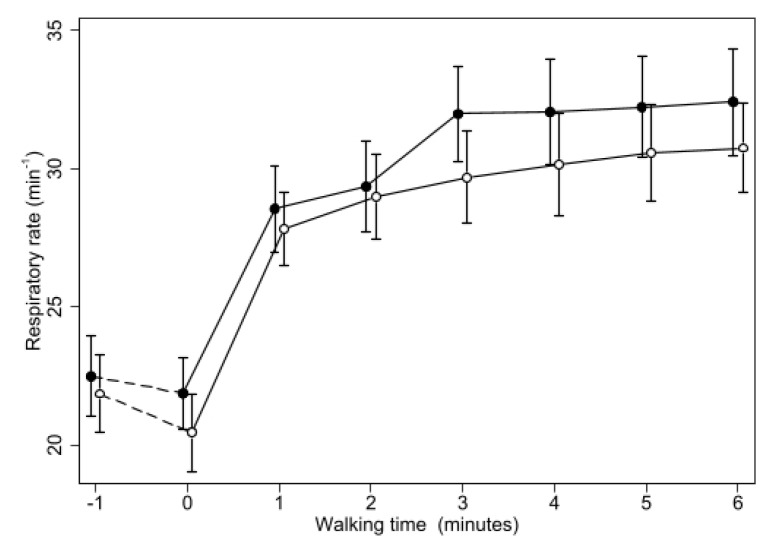
Changes of mean Respiratory Rate during 6MWT with oxygen or placebo. Same symbols as in Figure 1. The difference between the two treatments was not significant until minute 2 (when *p* was 0.03) and then was highly significant (*p* < 0.0005) at all the time points from minute 3 of the walking test.

Respiratory rate (Figure 4) also increased rapidly after patients started walking, but in this case the increase was similar during oxygen or placebo, and the difference between the two treatments was not significant during the first two minutes of walking. Afterwards, RR during placebo increased more than during oxygen (*p* < 0001 at all the time points from the third to the sixth minute of walking, *p* = 0.012 for trend over time).

**Figure 5:**
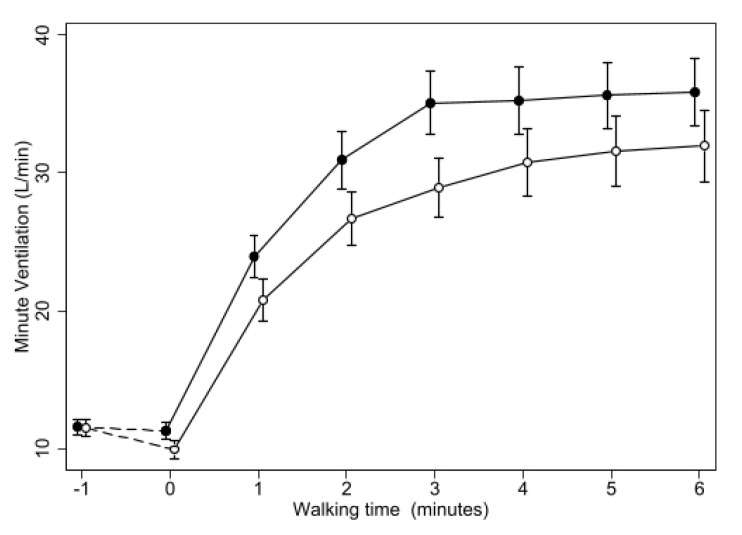
Changes of mean Minute Ventilation during 6MWT with oxygen or placebo. Same symbols as in Figure 1. The difference between the two treatments was highly significant (*p* < 0.0005) at all the time points from minute 1 of the walking test.

Minute ventilation (Figure 5) did increase steadily during walking with both treatments, but less so with oxygen, resulting in a highly significant difference (*p* < 0.001) at all the time points, without any significant change in the difference between the two treatments over time.

No significant effect of the order of treatments was found in any of the above analyses.

## 4. Discussion

In the present study, we found that patients with pulmonary fibrosis who were normoxic at rest and hypoxemic while walking, show a significant improvement in respiratory pattern and cardiovascular response to exercise when they receive oxygen during the 6 min walking test, compared to placebo. When on air, these subjects reached a peak tidal volume in the first minutes of the test that remained stable for the rest of the test. Respiratory rate showed a similar increase in the first few minutes and then remained constant.

When on oxygen, however, these patients initially showed a slower increase in tidal volume to meet their increased demand for oxygen. Beyond a certain limit, RR increased less than on placebo. As a result, VE remained lower during the whole 6MWT on oxygen than on air. Furthermore, the increase in HR induced by exercise was significantly reduced during oxygen.

We could not demonstrate a direct correlation between the reduction in dyspnoea and the reduced request for cardiovascular and respiratory effort in individual patients, probably because of the small number of patients compared with the variability of the measurements, but the fact that we could observe a significant decrease in end of test dyspnoea and an increase in the distance walked during treatment with oxygen, strongly suggests that it contributed to the improvement of exercise through those mechanisms. This explanation is consistent with our current knowledge on the mechanism of exercise impairment in patients with fibrotic ILDs, which is attributed to a complex interplay of lung mechanics, gas diffusion, vascular, cardiac, muscular and psychological factors [9,11].

By preventing oxygen desaturation, ambulatory oxygen reduces the additional burden imposed by the poor lung gas diffusion on ventilation and circulation and possibly improves muscle oxygenation, while other factors (lung mechanics, muscle conditioning, psychological factors) are not or hardly affected. Indeed, in our patients, mean walked distance expressed as percent of predicted changed from 73% (64–83) on placebo to 79% (71–87) on oxygen, thus remaining on average below the normal value. However, while pharmacological treatments are available to slow disease progression in these patients [16], no treatment is available to them to improve exercise tolerance, except ambulatory oxygen and pulmonary rehabilitation [17], which are not mutually exclusive and could be combined [18], so even a partial result is clinically relevant. Due to the requirements of our experimental settings, we had to use a walking circuit of 20 m, slightly shorter than the recommended length of 30 m [14]. This may have increased the opportunity to rest at turnings, as suggested by the reduced increase in fatigue reported at the end of test. It did not prevent the increase in respiratory and cardiovascular burden, however, while allowing to avoid the use of trolleys with portable gas canisters, which have been shown to increase the burden of exercise in these patients [19].

Few studies have yet evaluated the advantages and disadvantages of Spiropalm and its possible applications in clinical practice. De Martino et al. compared the Spiropalm-equipped 6MWT with the conventional one, finding no significant differences. In the same study, they also tried to compare these results with performance in the cardiopulmonary exercise test but found no overlap between these two functional tests [20].

We found that the sealed mask used with the Spiropalm spirometer does impose considerable discomfort on our patients, which could have impaired the performance of walking and interfered with the report of the end-of-test dyspnea. Apart from this, we found the instrument to be reliable and fit to the task. In future studies, however, less invasive instruments, such as wearable respiratory monitors [21], would be preferable.

Nevertheless, the experimental setting during oxygen and placebo was identical, and whatever limit might have been imposed, it could only have reduced the size of the estimated effect without introducing bias.

Our study confirms previous observations, including those by Ora J et al., who showed that the effect of ambulatory oxygen was significantly greater in ILD patients who were desaturated during physical exertion but not at rest. In fact, oxygen supplementation during exercise increased exercise tolerance and reduced the perception of dyspnoea in ILD patients with exercise-induced hypoxemia [20].

Not all prior studies showed positive outcomes. Nishiyama et al. (2013) showed that 4 L/min oxygen supplementation did not improve dyspnoea after a standardized 6MWT or a 6 min free walk in 20 IPF patients without resting hypoxemia. Nor did oxygen therapy during exercise improve the distance walked, leg fatigue or heart rate [22].

However, the fact that we used a standardized method to tailor oxygen flow according to individual needs could explain the differences with our study, as some patients may require more than 4 L/min.

Not only is ambulatory oxygen generally underprescribed in interstitial lung diseases [3], and only recently found its way on expert recommendations [17], but often patients are reluctant to accept and use it [23], partly because it is difficult for them to understand its purpose and possible benefits. Our data, obtained in a familiar context such as walking, could be helpful to communicate to patients the expected benefits, in a setting of shared decision making [24].

In conclusion, this study showed that ambulatory oxygen, in addition to (and probably by) correcting hypoxaemia, does reduce the respiratory and cardiovascular effort required during walking in patients with fibrotic ILDs that desaturate on exercise, and this phenomenon could at least partially explain the benefit observed on quality of life in these patients.

## Figures and Tables

**Table 1:** At the end of each test, the patient was asked to quantify his/her dyspnoea and fatigue on a Borg scale.

% Male	85	(69.4–100.6)
Age (Yrs)	72.2	(68.4–75.9)
% History of smoking	70	(50–90)
BMI	27.2	(25.8–28.6)
% pred FVC	75.1	(68.5–81.6)
% pred FEV1	76	(67.4–84.6)
% FEV1/FVC	77.3	(73.7–80.9)
%pred TLC	73.5	(66.5–80.5)
% pred DLCO	72.2	(63.3–81)

**Table 2:** Summary data of the 6MWT are reported in this table.

	Placebo		Oxygen		Difference	
Distance Walked (m)	340	(301–379)	368	(337–399)	27.8	(2.4–53.2) *
Baseline dyspnoea	1.6	(0.7–2.5)	1.3	(0.5–2.1)	−0.2	(−0.5–0.1)
Baseline Fatigue	0.3	(0–0.6)	0.2	(−0.1–0.5)	−0.1	(−0.5–0.3)
Minimum oxygen saturation	82.3	(80.1–84.5)	92	(90.3–93.7)	9.7	(7.8–11.6) ***
Maximal Heart Rate	103	(96.2–109.8)	97.6	(92.2–103) **	−5.4	(−8.7–−2.1) **
Final dyspnoea	4.2	(3.6–4.8)	3.6	(2.8–4.4) *	−0.6	(−1.1–−0.1) *
Final Fatigue	1.1	(0.4–1.8)	0.8	(0.2–1.4)	−0.3	(−0.7–0.1)

* <0.05, ** <0.005, *** <0.0005.

## Data Availability

The data presented in this study are available upon request from the corresponding author.

## Abbreviations

Interstitial lung diseases (ILDs), idiopathic pulmonary fibrosis (IPF), forced vital capacity (FVC), forced expiratory volume in the first second (FEV1), total lung capacity (TLC), functional residual capacity (FRC), diffusing capacity of the lung for carbon monoxide (DLCO), high-resolution computed tomography (HRCT), usual interstitial pneumonia (UIP), six-minute walking test (6MWT), The University of California, San Diego Shortness of Breath Questionnaire (UCSDSOBQ), King’s Brief Interstitial Lung Disease (K-BILD) questionnaire, respiratory rate (RR), Tidal volume (V_T_), minute ventilation (VE).
